# Harnessing Intronic microRNA Structures to Improve Tolerance and Expression of shRNAs in Animal Cells

**DOI:** 10.3390/mps5010018

**Published:** 2022-02-10

**Authors:** Arjun Challagulla, Mark L. Tizard, Timothy J. Doran, David M. Cahill, Kristie A. Jenkins

**Affiliations:** 1 CSIRO Health and Biosecurity, Australian Centre for Disease Preparedness, Geelong, VIC 3219, Australia; arjun.challagulla@csiro.au (A.C.); Mark.Tizard@csiro.au (M.L.T.); Timothy.Doran@csiro.au (T.J.D.); 2 School of Life and Environmental Sciences, Deakin University, Geelong, VIC 3217, Australia; david.cahill@deakin.edu.au

**Keywords:** avian, RNAi, promoters, transgenic, miRNAs, shRNA

## Abstract

Exogenous RNA polymerase III (pol III) promoters are commonly used to express short hairpin RNA (shRNA). Previous studies have indicated that expression of shRNAs using standard pol III promoters can cause toxicity in vivo due to saturation of the native miRNA pathway. A potential way of mitigating shRNA-associated toxicity is by utilising native miRNA processing enzymes to attain tolerable shRNA expression levels. Here, we examined parallel processing of exogenous shRNAs by harnessing the natural miRNA processing enzymes and positioning a shRNA adjacent to microRNA107 (miR107), located in the intron 5 of the Pantothenate Kinase 1 (PANK1) gene. We developed a vector encoding the PANK1 intron containing miR107 and examined the expression of a single shRNA or multiple shRNAs. Using qRT-PCR analysis and luciferase assay-based knockdown assay, we confirmed that miR30-structured shRNAs have resulted in the highest expression and subsequent transcript knockdown. Next, we injected Hamburger and Hamilton stage 14–15 chicken embryos with a vector encoding multiple shRNAs and confirmed that the parallel processing was not toxic. Taken together, this data provides a novel strategy to harness the native miRNA processing pathways for shRNA expression. This enables new opportunities for RNAi based applications in animal species such as chickens.

## 1. Introduction

The discovery of the RNA interference (RNAi) mechanism in eukaryotes has profoundly enabled our ability to understand the function of genes in living cells and organisms [1]. RNAi is regularly used to knockdown endogenous messenger RNA (mRNA) or exogenous targets such as viral RNA using small interfering RNAs (siRNAs) or short hairpin RNAs (shRNA), both of which rely on the endogenous microRNA (miRNA) pathway [2]. The siRNAs are short synthetic RNAi effector molecules which do not require any processing and enter the RNAi pathway at the RNA inducing silencing complex step [3]. In contrast, shRNAs are hairpin-like RNA structures that mimic miRNA structures and require sequential cleavages by cellular miRNA processing enzymes to form mature siRNAs [4]. Given the challenges associated with delivery of siRNAs in vivo, the use of transgenic approaches to stably introduce shRNAs into the genome to facilitate intracellular synthesis of siRNAs is more appealing [5]. 

Expression of shRNAs using exogenous polymerase (pol) III promoters such as U6 in a DNA vector is an efficient and convenient approach to induce RNAi in living cells or organisms. Despite the significant body of research on identification and characterisation of species-specific pol III promoters in vitro [5,6,7,8,9,10], there remains an incomplete understanding of their functionality in vivo. Previous studies have reported that stable expression of shRNAs in vivo can cause severe toxicity issues in mice in multiple tissue types including the heart [11], brain [12,13] and liver [14]. These toxicity issues are primarily attributed to the saturation of the natural miRNA pathway with exogenous shRNAs. Because miRNAs play a dynamic role in regulation of gene expression where they act to control cellular and metabolic pathways in a spatiotemporal manner, saturation of the endogenous miRNA pathway can perturb miRNA-dependent regulation of biological processes [15]. Although weaker promoters such as H1 [16] or tissue specific pol II [17] promoters can be used to reduce shRNA-associated toxicity, they can also result in expression levels which are suboptimal for a biological outcome for some applications. Additionally, constructs encoding shRNA under the control of promoters and regulatory sequences delivered by either viral [14] or non-viral [18] vectors often result in random integration of multiple copies into the genome. These approaches can have downstream consequences not only to the biology of the animal but to the acceptance, by regulatory bodies and consumers, of the subsequent genetically modified (GM) animal. This is a hurdle for adoption of RNAi in livestock species and gene technology regulatory authorities will apply stringent controls and require a detailed safety assessment for GM animal food products prior to their approval. 

The aim of this study to investigate if expression of a promoter-less shRNA can be achieved by the natural miRNA processing pathways when positioned adjacent to an endogenous miRNA. We selected miR107 as our candidate miRNA for parallel processing of either a single or multiple shRNAs and confirmed knockdown of targeted genes. Finally, we demonstrated that the expression levels achieved from this approach are tolerated by the primordial germ cells (PGCs) of developing chicken embryos suggesting that a germline-modified chicken that carries this shRNA transgene could be successfully generated.

## 2. Materials and Methods

### 2.1. Primers 

All primers used in this study were synthesised as standard polymerase chain reaction (PCR) grade with desalt purification (Geneworks, Thebarton, Australia). The single-stranded oligos used for shRNAs were synthesised with high-performance liquid chromatography purification. The sequences of all primers used in this study are listed in Supplementary Table S1.

### 2.2. Construction of pGFP-Intron (pGI) Vector

To generate the pGI vector, we performed the molecular cloning in six steps. In step 1, a SmaI restriction enzyme (RE) site was introduced into the coding sequence of the enhanced green fluorescent protein (eGFP) coding sequence encoded on the peGFP-N1 (Clonetech, Mountain View, CA, USA) vector by PCR amplifying the eGFP coding sequence into 2 sections using the following primers: GFP_fwd+SmaI_rev (for Section 1) and SmaI_fwd+GFP_rev (for Section 2). In step 2, the two eGFP sections were used as the template for another round of PCR, using GFP_fwd and GFP_rev primers to amplify the full mutant eGFP product containing the newly introduced SmaI site. In step 3, the eGFP-SmaI fragment was directionally cloned into SalI and NotI RE sites of the original pEGFP-N1 backbone vector. In step 4, the Intron 5 of pantothenate kinase 1 (PANK1) (3595 bp) was PCR amplified from genomic DNA of white leghorn chicken using primers Intron_107_fwd and Intron_107_rev using Platinum PCR supermix (Invitrogen, Waltham, MA, USA). In step 5, we introduced BamHI (133 nt downstream of miR107) and KpnI (391 nt down stream of miR107) RE sites using the primers BamHI_int_fwd, BamHI_int_rev, KpnI_int_fwd and KpnI_int_rev. In step 6, the modified intron was directionally cloned into the SmaI site of eGFP sequence to generate the peGFP-intron (referred to as pGI from now) vector. 

### 2.3. Cloning of shRNAs into pGI Vector

The siRNAs targeting polymerase basic protein 1 (PB1) [19], nucleoprotein (NP) and nuclear export protein & non-structural protein 1 (NS1/NEP will be referred to as NS) of influenza A genome are listed in Supplementary Table S2. For single shRNA expression, shRNAs were designed to have either the Brummelkamp (BK) loop [4] or miRNA30 (miR30) backbone structure (Figure S1). Additionally, miR30 modelled shRNAs were designed to contain mismatch or bulges in the stem and the loop sequence to reflect natural miR30 structure. The single BK-PB1 shRNA was generated using the primers PB1_BK_fwd and PB1_BK_rev, while the miR30-PB1 shRNA was generated using the primers miR30_PB1_fwd and miR30_PB1_rev. To clone a single shRNA (BK-PB1 or miR30-PB1) coding sequence into the pGI vector (Figure S2), complementary oligos containing BamHI and KpnI RE overhangs were annealed using 2 µL forward strand (1 µg/mL) and 2 µL reverse strand (1 µg/mL) in 46 µL of annealing buffer. The oligo mix was heated to 90 °C for 1 min, followed by a 2-h incubation step at room temperature. The annealed oligos were diluted at 1:10 ratio and directionally cloned into the BamHI and KpnI RE sites of the pGI vector to generate pG1-BK-PB1 and pGI-miR30-PB1, respectively. 

To develop a multiple shRNA expression cassette, we modified three loop sequences downstream of miR107; position 1520 nt to 1522 nt, position 1586 nt to 1588 nt, position 1648 nt to 1655 nt of intron 5 of the chicken PANK1 gene and replaced it with three miR30 structured shRNAs (Figure S3). The modified intron containing the three shRNAs (size: 466 bp) with introduced BamHI and KpnI RE sites was synthesised (Life Technologies, Carlsbad, CA, USA) (Figure S4) and cloned into BamHI and KpnI sites of the pGI base vector to generate the pGI-PB1-NP-NS vector. 

To examine the shRNA-mediated knockdown of target genes, a dual luciferase assay was performed using psiCHECK2 (Promega, Madison, WI, USA) vector carrying a section of the target gene that contains the siRNA specific sequence (Promega). To generate psiCHECK2 vectors encoding target genes, we PCR amplified approximately 500 bp of sections of PB1 gene using PB1_luc_fwd and PB1_luc_rev, NP gene using NP_luc_fwd and NP_luc_rev and NS gene using NS_luc_fwd and NS_luc_rev. The amplified PCR amplicons were ligated into XhoI and NotI restriction sites in the psiCHECK2 vector to produce psiCHECK-PB1, psiCHECK-NP and psiCHECK-NS, respectively. 

### 2.4. Cell Culture and Transfections

DF1 cells (American Type Culture Collection number: CRL-12203) were grown in cell culture as previously described [20]. To assess single shRNA expression from the pG1 vector, DF1 cells were transfected with 3 ug of desired pGI-BK-PB1 or pGI-miR30-PB1 vector and total RNA was extracted 48 h post-transfection. To perform luciferase knockdown assays for the single shRNA expression vector, DF1 cells were transfected with 1.5 µg of pGI-BK-PB1 or pGI-miR30-PB1 vector and 1.5 µg of psiCHECK2 containing either the PB1, NP or NS targeted gene section. For multiple shRNA work, DF1 cells were transfected with 1.5 µg of pGI-PB1-NP-NS vector and 1.5 µg of psiCHECK2 containing either the PB1, NP or NS targeted gene section. Both plasmids were complexed with 8 µL of lipofectamine 2000 (L2000) and added to each well. Knockdown cells were harvested at 48 h post-transfection and luciferase expression was measured using the dual-luciferase reporter assay (Promega) and GloMax multi detection system (Promega) as per the manufacturer’s instructions. The ratio of renilla luciferase to firefly luciferase was obtained and normalised. 

### 2.5. RNA Extraction

Total RNA from DF1 cells transfected with pGI-BK-PB1 or pGI-miR30-PB1 was extracted at 48 h post-transfection using Trizol Reagent (Invitrogen) according to the manufacturer’s instructions. Briefly, 10 µg of glycogen (Invitrogen) was added to the aqueous phase and 80% ethanol was used for the wash step to enhance the precipitation of small RNAs. RNA pellets were resuspended in 20 µL of nuclease-free (NF) water. Prior to reverse transcription, RNA samples were treated with RQ1 DNase (Promega) according to the manufacturer’s instructions. 

### 2.6. Polyadenylation of RNA, cDNA Synthesis and qRT-PCR

The extracted total RNA was subjected to polyadenylation and cDNA synthesis as previously described [21]. Briefly, polyadenylation of RNA was carried out using approximately 1 µg of total RNA, 0.25 µL (150 U) of yeast poly (a) polymerase (PAP) (catalogue no. 74225; USB corporation), 4 µL 5x PAP buffer and 1 µL of 10 nM rATP (Ambion, Austin, TX, USA) and NF water (Promega) to a final volume of 20 µL. Reactions were incubated at 37 °C for 30 min and then 95 °C for 5 min. To perform cDNA synthesis, we used the SuperScript III First-Strand Synthesis System (Invitrogen) and each reaction contained 4 µL of polyadenylated total RNA, 3 µL of modified oligo-dT primer (miR-PTA) and 1 µL of annealing buffer mix and followed manufacturer instructions. 

Analysis of shRNA or miRNA expression by qRT-PCR was performed as previously described [21]. In brief, a universal reverse primer PAM-URP to recognise miR-PTA sequence and miRNA (miR107 or miR26a) or shRNA (PB1 siRNA) specific forward primers were used to measure expression levels. The chicken ribosomal small RNA (5S rRNA) was used as the reference control. Each reaction contained 2 µL of 1:50 diluted cDNA in NF water, 10 µL SYBR Green PCR Master Mix (Applied Biosystems, Waltham, MA, USA), 0.8 µL of each primer (final concentration 200 nM) and NF water to 20 µL final volume. All samples were analysed in triplicate in 96 well MicroAmp PCR plates (Applied Biosystems) using the StepOnePlus real-time PCR system (Applied Biosystems). Cycle settings were 94 °C, 10 min; 94 °C, 15 s and 60 °C, 1 min (40 cycles). The delta–delta Ct method was used to calculate the relative fold difference between samples. To quantify miR107 or miR26a levels in cells or tissues, expression levels of the respective miRNAs were normalised to the 5S rRNA reference control and calculated as a fold difference relative to the untransfected control (cell work) or brain (tissue work). To quantify PB1 siRNA levels in cells transfected with pG1 vectors, expression levels of miR30-PB1 were made relative to BK-PB1 to quantify the levels of PB shRNAs in transfected DF1 cells. 

### 2.7. miniTol2 Plasmids

The miniTol2 transposon system used in this study was as previously published [22]. In brief, the miniTol2 system is a two-plasmid system of which one plasmid contains the terminal Tol2 sequences flanking the transgene insert, while the second plasmid contains the transposase coding sequence under the control of the CAGGS promoter (designated as pTrans). Transfection of both the transgene and pTrans plasmids into cells allows stable insertion of the transgene into the genome which is catalysed by the transposase enzyme expressed from the pTrans vector. To assess if the shRNAs levels expressed from the pGI vector caused any toxicity issues in chicken primordial germ cells (PGCs), we developed a Tol2 transposon vector carrying intron 5 of the PANK1 gene (referred to as pTol-GI) to stably transfect PGCs. To construct the pTol-GI vector, the “EG-intron-FP” fragment from the base pGI vector was excised with NotI and XhoI REs and cloned distal to the CAGGS promoter within the Tol2 vector. The Poly-A sequence was PCR amplified using primers Poly-A_Fwd and Poly-A_Rev from the base pGI vector and cloned into the Not1 RE site to generate the final pTol-GI vector. To generate the transposon vector carrying both the intron and miR30-based multiple shRNAs(pTol-GI-PB1-NP-NS), the oligo-synthesised 466 bp intron sequence (modified intron fragment with three shRNAs) was cloned into BamHI and KpnI RE sites of intron 5 region within the pTol-GI vector to generate pTol-GI-PB1-NP-NS vector. 

### 2.8. Assessment of Toxicity in Chicken Embryos 

To assess the toxicity of the shRNA expression using this system, we injected the pTol-GI-PB1-NP-NS vector into dorsal aorta of Hamburger and Hamilton (HH) stage 14–15 chicken embryos as previously described [23]. Briefly, 0.6 µg of pTol-GI-PB1-NP-NS vector and 1.2 µg of pTrans vectors were mixed with 45 µL of OptiPRO (Invitrogen) and incubated at room temperature for 5 min. In another tube, 3 µL of L2000 CD (Invitrogen) was added to 45 µL of OptiPRO and incubated for 5 min. These two solutions were mixed and incubated at room temperature for 20 min to allow complex formation, prior to injecting into embryos. Approximately 1–2 µL of complex was injected into the dorsal aorta HH stage 14–15 chicken embryo. To examine eGFP fluorescence, gonads from HH stage 40 recipient embryos were dissected and viewed under a fluorescence microscope for eGFP expression.

### 2.9. Statistical Analysis

All statistical analyses were carried out using GraphPad Prism software. Statistical comparisons of miR107 and shRNA expression from qRT-PCR data was performed using a one-way Anova with a Dunnett’s multiple comparison post-test. Statistical comparison of luciferase knockdown assay for the single shRNA vector was performed via one-way Anova with a Dunnett’s multiple comparison post-test. Statistical comparison of luciferase knockdown assay for the multiple shRNA vector was performed individually for each target by a two-tailed parametric *t*-test with Welch’s correction. 

## 3. Results

### 3.1. Identification of a Universally Expressed Intronic miRNA 

**Figure 1:**
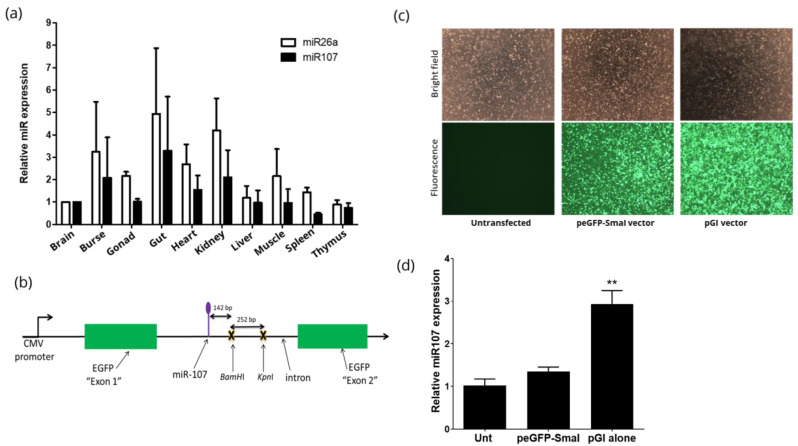
Construction and validation of the peGFP-intron (pGI) vector in chicken DF1 cells. (**a**) Analysis of miR107 and miR26a expression in various tissues from HH stage 44 chicken embryos. miRNA expression was quantified using qRT-PCR for miR26a (white bars) and miR107 (black bars) in different tissues of chicken embryos. The expression levels of corresponding miRNAs was normalised to 5S reference control and calculated as a fold difference relative to the brain sample. All samples were analysed in triplicate and error bars represent standard deviation (SD). (**b**) Schematic depiction of the pGI vector with introduced BamHI and KpnI restriction enzyme sites adjacent to miR107 within the cloned intron 5 of the PANK1 gene (**c**) Analysis of eGFP expression in cells transfected with peGFP-SmaI, pGI vector or untransfected control. Bright field (**top row**) and fluorescence (**bottom row**). (**d**) Analysis of miR107 expression levels in cells transfected with pEGFP-SmaI, pGI or untransfected control (unt) at 48 h post-transfection using qRT-PCR. miRNA expression was normalised to 5S reference control and calculated as a fold difference relative to the untransfected control. Error bars represent SD. Asterisks indicate statistical significance: ** *p* < 0.005.

To examine if an exogenous shRNA could be expressed by utilising natural miRNA processing pathways, we began by identifying a miRNA that is highly expressed in different tissues of the chicken. Previously, our laboratory had carried out deep sequencing of RNA from chicken embryos [24] and analysis of this data identified two potential candidates; miR107 which was 35th on the RNAseq count and miR26a which was 56th on the RNAseq count. The reads for miR107 ranged from 36813–26822 while the reads for miR26a ranged from 15108–19010. Next, we consulted the gallus expression in situ hybridisation analysis (GESHIA) website [25], which has in situ hybridisation data for all known chicken miRNAs. The GESHIA website showed widespread expression of both miR26a and miR107 except in the heart of the Hamburger–Hamilton (HH) stage 25 chicken embryos (data not shown). To confirm the expression data, we carried out qRT-PCR analysis on total RNA extracted from organs of HH stage 44 chicken embryos and found that both miR26a and miR107 were expressed in all examined tissues, including the heart (Figure 1a). Prior to selecting the candidate miRNA for parallel processing, we examined the genomic location at which these miRNAs were located. The location of miR107 is in a ~3.5 kb intron of pantothenate kinase 1 (PANK1) (Gene ID: 423792), this essential gene is involved in the Pantothenate and Coenzyme A (CoA) biosynthesis pathway [26]. While miR26a is in a ~10 kb intron of carboxy-terminal domain small phosphatase-like protein (Gene ID: 408252). Due to its more consistent expression and location within a smaller intron of an essential gene, we selected miR107 for the parallel processing approach. 

### 3.2. Construction and Characterisation of peGFP-Intron 

To examine the parallel processing approach utilising the natural miRNA pathway, we first developed and characterised the peGFP-Intron vector (referred to as pGI) that contains miR107, by inserting the ~3.5 kb intron 5 of the PANK1 gene into the peGFP-N1 base vector. For this purpose, we introduced a unique SmaI RE site into the eGFP coding sequence of peGFP-N1 vector to generate peGFP-SmaI vector. Next, intron 5 from the PANK1 gene including the acceptor and donor sites was directionally cloned into the SmaI RE to generate the pGI vector (Figure 1b), which mimics the genomic location of miR107 between the exons in the PANK1 locus. Transfection of pGI into cells will produce GFP expression if the introduced intron is spliced out of the transcript and the two eGFP exons are joined to enable translation. To test this, DF-1 cells were transfected with pGI (test), peGFP-SmaI (positive control for eGFP expression), or left untransfected (negative control for eGFP expression). Fluorescence microscopy analysis confirmed eGFP expression in both peGFP-SmaI and pGI transfected cells after 48 h post-transfection, indicating that the introduced intron was successfully spliced from the transcript (Figure 1c). Subsequently, the expression levels of miR107 were quantified by qRT-PCR and we observed a 3-fold increase (*p* < 0.001) in cells transfected with the pGI vector compared to cells transfected with the peGFP-SmaI or untransfected cell control (Figure 1d). 

### 3.3. Expression of shRNAs from the pGI Vector

**Figure 2:**
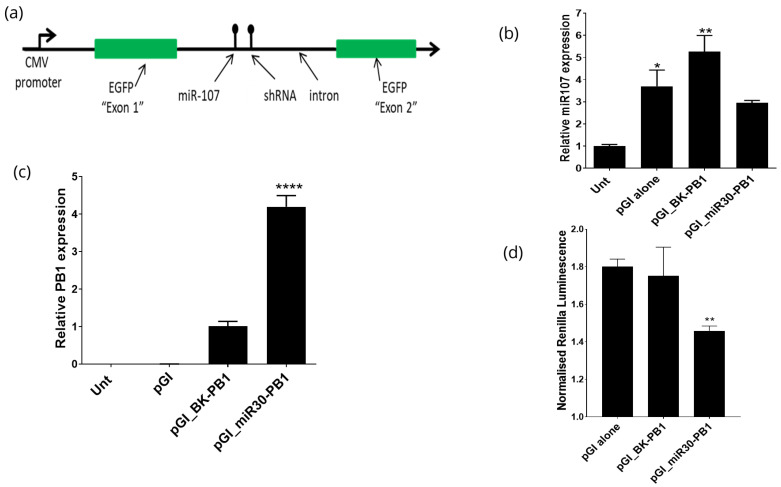
Characterisation of single shRNA expression from the pGI vector. (**a**) Schematic of the pGI vector cloned with a single shRNA (BK-PB1 or miR30-PB1) adjacent to miR107 within the intron 5. (**b**) Analysis of the effect on miR107 expression levels in cells transfected with different pG1 vectors. DF1 cells were transfected with either pGI alone, pGI-BK_PB1, pGI-miR30_PB1 or left untransfected (unt). The level of miR107 expression was normalised to 5S reference control and calculated as a fold difference relative to the untransfected control. (**c**) Analysis of PB1 siRNA levels in DF1 cells transfected with pEGFP-SmaI, pGI_BK-PB1, pGI_miR30-PB1 or untransfected control. The levels of PB1 siRNA were normalised to the 5S reference control and calculated as a fold difference relative to cells transfected with the pGI_BK_PB1 vector. (**d**) Luciferase reporter assay to assess knockdown efficiency. A section of the PB1 gene containing the siRNA target sequence was cloned into the psiCHECK2 vector. DF1 cells were co-transfected with psiCHECK-PB1 and pGI_BK-PB1 or pGI_miR30-PB1. Values represent mean ratios of Renilla: Firefly luciferase ±SD from *n* = 3, measured in triplicate and representative of 3 independent experiments. Asterisks indicate statistical significance: * *p* < 0.05, ** *p* < 0.005 and **** *p* < 0.0001.

To examine if parallel processing of an exogenous shRNA can be achieved by positioning a single shRNA next to miR107, the intron within the pGI vector was modified to contain BamH1 and KpnI restriction enzyme (RE) sites to subsequently clone the shRNA sequences (Figure 1b). The influenza PB1 siRNA (hereafter referred to as PB1) was chosen as a target sequence to generate shRNA structures using a classic brummelkamp (BK) loop or miR30 structure to generate BK-PB1 and miR30-PB1 shRNAs, respectively (Figure S1). The designed shRNAs were individually cloned into BamH1 and Kpn1 RE sites of the pGI vector to generate pGI_BK-PB1 and pGI_miR30-PB1, respectively (Figure 2a). Following the transfection of these vectors individually into DF1 cells, eGFP expression was confirmed at 48 h post-transfection, indicating that the addition of the shRNA into the pG1 vector did not interfere with intron splicing (data not shown). Total RNA extracted from the transfected cells was used to quantify expression levels of miR107 and PB1 shRNA. The miR107 levels in cells transfected with pGI alone, pGI_BK-PB1 and pGI_miR30-PB1 were elevated compared to untransfected cells (Figure 2b). Between the different pGI vector transfected DF1 cells, we observed significantly higher levels of miR107 in pGI_BK-PB (*p* < 0.001) compared to the base pGI vector or pGI_miR30-PB (Figure 2b). The levels of the PB siRNA in cells transfected with the miR structured shRNA (pGI_miR30-PB1) were approximately 4.5-fold more than those transfected with the BK loop shRNA (pGI_BK-PB1) (*p* < 0.0001) (Figure 2c). Next, to assess if the shRNAs were being processed and could mediate targeted knockdown of the PB1 gene of influenza A virus, the vector psiCHECK2 containing the PB1 (psiCHECK-PB1) target region was used. DF1 cells were co-transfected with the pGI _BK-PB1 or pGI_miR30-PB1 and psiCHECK-PB1, followed by measurement of firefly and renilla luminescence at 48 h post transfection. Results indicated that cells transfected with pGI_miR30-PB significantly (*p* < 0.01) reduced the levels of normalised renilla luminescence compared to base pGI vector or pGI_BK-PB (Figure 2d) which indicates target gene knockdown. Combined the qRT-PCR and knockdown assay confirm the expression and processing of the introduced shRNAs. 

### 3.4. Expression of Multiple shRNAs from the pGI Vector

**Figure 3:**
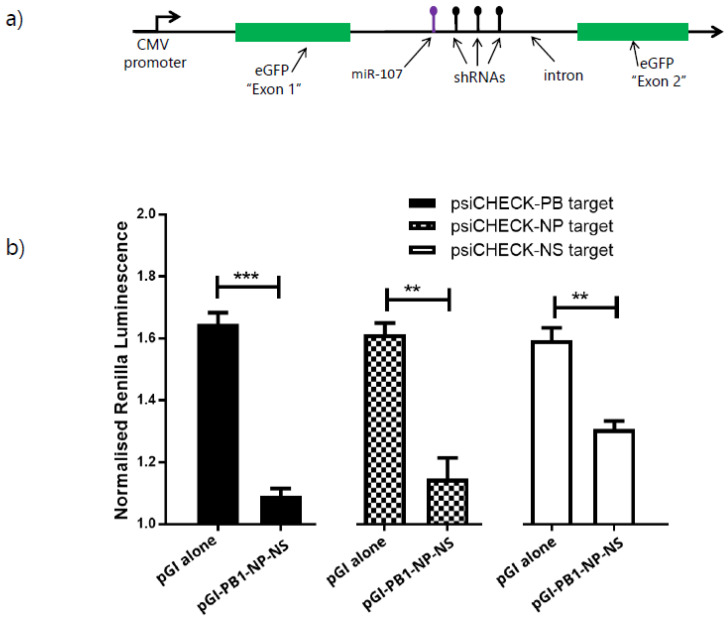
Validation of multiple shRNAs expressed from the pGI vector (**a**) Schematic of the pGI vector with three miR30 structured shRNAs cloned into intron 5 of the pGI vector. (**b**) Dual luciferase reporter based knockdown analysis of the corresponding target gene sections of PB1, NP or NS genes. DF1 cells were co-transfected with psiCHECK2 (containing section of either PB1, NP or NS gene) and pG1 alone (control) or pGI-PB1-NP-NS. Transfected cells were harvested two days post-transfection and a dual luciferase reporter assay was performed. Values represent mean ratios of Renilla: Firefly luciferase ± SD. Asterisks indicate statistical significance: ** *p* < 0.005, *** *p* < 0.0005.

When targeting an exogenous agent such as a virus, the application of multiple shRNAs can overcome any limitations that arise due to the emergence of escape mutants [27,28]. To assess whether multiple shRNAs can be processed by the parallel processing approach we expressed three miR30 structure based shRNAs targeting influenza genes (PB1, NP and NS). These were positioned downstream of miR107 within intron 5 of the PANK1 gene (Figure S3). The synthesised intron fragment with three shRNAs was positioned downstream of miR107 within the pGI vector to generate pGI-PB1-NP-NS (Figure 3a). Following the transfection of pGI-PB1-NP-NS into DF1 cells, fluorescent microscopy analysis confirmed eGFP expression in transfected cells (data not shown), indicating that the addition of the synthesised fragment with three shRNAs into the pGI vector did not interfere with intron splicing. To assess if the shRNAs expressed from pGI-PB1-NP-NS could mediate targeted knockdown, DF1 cells were co-transfected with either pGI (negative control) or pGI-PB1-NP-NS (test) and one of the three psiCHECK2 constructs (psiCHECK-PB1, psiCHECK-NP or psiCHECK-NS target). The renilla luciferase and firefly luciferase levels were measured 48 h post transfection. Results indicated that the expression of multiple shRNA from the pGI-PB1-NP-NS vector significantly reduced the levels of normalised renilla luminescence compared to the pGI vector alone for all the corresponding targets (Figure 3b) indicating that the three shRNAs expressed from the intron have mediated targeted knockdown.

**Figure 4:**
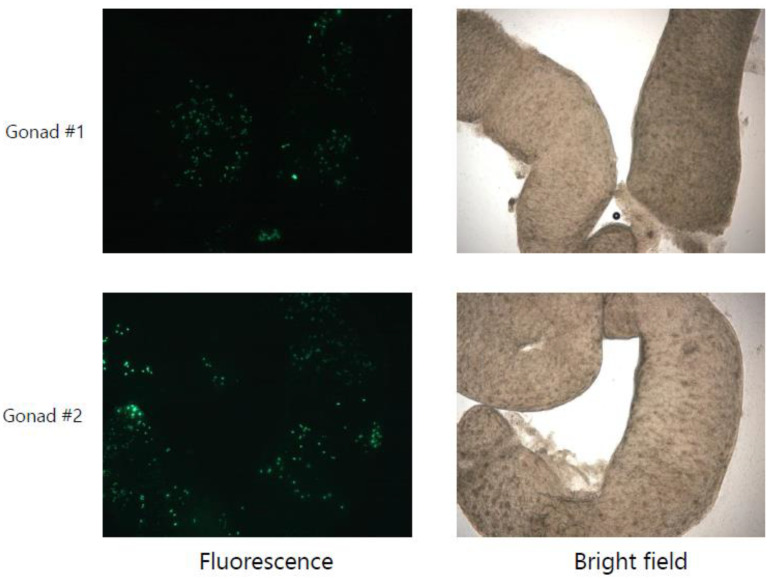
Analysis of gonads from chicken embryos injected with the pTol-GI-PB1-NP-NS vector. The eGFP and bright field images of gonads dissected from HH stage 40 chicken embryos that were injected with pTol-G1-PB1-NP-NS at HH stage 14–15.

As we were able to show expression and targeted knockdown from single and multiple shRNAs in the pGI vector, we next examined if stable introduction of an “EG+Intron-PB1-NP-NS+FP” transgene would cause any toxic effects in primordial germ cells (PGCs) in developing chicken embryos. We constructed a Tol2 transposon vector to carry the EG+Intron-PB1-NP-NS+FP transgene under the control of a CAGGS promoter (hereafter referred to as pTol-GI-PB1-NP-NS). Co-transfection of pTrans and pTol-GI- PB1-NP-NS vectors results in the stable insertion of the “EG+intron-PB1-NP-NS+FP” sequence into the genome of the PGCs. We intravenously injected 10 stage 14–15 (HH) embryos with pTol-GI-PB1-NP-NS and pTrans formulated with L2000 CD. Nine out of the 10 injected embryos survived to HH stage 40, and gonads from these embryos were analysed under a fluorescence microscope. The eGFP was observed throughout the gonads of all dissected gonads (Figure 4), indicating that the PGCs tolerate the expression levels achieved from the integrated intron-PB1-NP-NS transgene. 

## 4. Discussion

We have demonstrated a novel shRNA expression strategy that utilises the nuclear regulatory and transcript processing enzymes for miRNAs. In this case we achieved parallel processing alongside miR107, without the need for an exogenous promoter or other regulatory elements. This miRNA is located within intron 5 of the PANK1 gene, a gene that is involved in Pantothenate and Coenzyme A (CoA) biosynthesis. CoA is a ubiquitous and essential cofactor that plays a central role in the metabolism of carboxylic acids, including short- and long-chain fatty acids and the oxidation of pyruvate in the citric acid cycle [26]. This gene is essential for all cells and, therefore, if the introduction of an exogenous shRNA within intron 5 interfered with gene function, then any detrimental effect would be apparent. Additionally, miR107 is a highly expressed miRNA in all organs of the developing chicken embryo that we have examined, and this is important for our strategy as we aim to achieve robust shRNA expression levels. Hence, positioning an exogenous shRNA adjacent to miR107 using genome engineering tools could potentially enable ubiquitous and tolerable shRNA expression levels in vivo, avoiding the recognised problems associated with overexpression of shRNAs in higher order eukaryotes.

The significantly higher levels of mature PB1 siRNA in cells transfected with pGI_miR30-PB1 compared to pGI_BK-PB1 suggests that miR30 modelled shRNAs structures served as a more efficient substrate for Drosha/Dicer-mediated cleavages. Previously, expression of shRNAs using the BK loop and under the control of pol II [29] or pol III [4] promoters have shown robust expression levels. However, a major caveat of using conventional stem-loop structures is that these structures undergo imprecise Drosha/Dicer-mediated processing and increase the risk of off-target effects [30]. In contrast, our parallel processing approach relies on natural miRNA processing enzymes, and we have demonstrated that miR30-PB1 structured shRNAs facilitate increased levels of expression compared to BK-PB1 structured shRNAs. Additionally, the dual luciferase assay showed that potent knockdown was observed in cells transfected with pGI_miR30-PB1 compared to pGI_BK-PB1. This confirmed that the levels and processing of shRNAs with a miR30 backbone are sufficient to elicit knockdown of the target gene. This finding agrees with previous reports suggesting that more potent gene silencing can be achieved using miRNA structured shRNAs compared to conventional shRNA structures [13,31,32]. It is worth noting that the chicken miR30 loop differs to human miR30 loop by two nucleotides. Previously, Hinton et al. [33] demonstrated that the use of human and chicken native miR30 loops improved silencing efficacy compared to other loop sequences but did not find a significant difference between the human and chicken miR30 loops. However, it should be noted that although Hinton et al. used full length miR30 loop sequences they did not incorporate any bulges into the seed sequence which we have done in our study.

It is widely acknowledged that combinatorial targeting of viral genes can limit emergence of escape mutants arising from siRNA induced selection pressure [28,34,35]. To this end, we explored the possibility of expressing three shRNAs using our parallel processing approach. To ensure efficient Drosha/Dicer mediated cleavage of exogenous shRNAs, we replaced three loop projections within intron 5 for minimal secondary structure distortions (Figure S3). All three miR30 structured shRNAs from the pGI-PB1-NP-NS vector demonstrated knockdown of target genes in DF1 cells in a luciferase assay. Additionally, by using a Tol2 system we demonstrate that the expression of multiple shRNAs behind miR107 were tolerated in ovo as indicated by the presence of GFP positive PGCs in the gonads following direct injection into the bloodstream. Importantly, this assay showed that expression levels and processing of the exogenous shRNAs was not having a toxic effect as has been previously reported when pol II promoters are used. 

Directions for future studies could involve investigating different insertion sites aside from the miR107 locus, for example, positioning of antiviral shRNAs on an innate immune-response gene. Indeed, this strategy could benefit antiviral intervention by concurrent expression of antiviral shRNAs and the innate immune system in a tissue-specific manner to control viral infection in transgenic animals. An advantage of this approach is most immune-related genes are inactive during early embryonic development reducing the risk of shRNA-associated toxicity. Here, we have characterised the ability to achieve parallel processing using an intronic miRNA, it will be important in future studies to confirm this ability using microRNAs coded for in 3′UTRs. 

Disease resistance in animal production is becoming an increasingly important issue in food security, trade and zoonotic spread of viral diseases [36]. The emergence of genome manipulation technologies such as CRISPR/Cas9 allows precise integration of transgenes into the genome for trait enhancement [37]. In the case of RNAi technology, CRISPR/Cas9 can be used to harness the natural processes of expression of small interfering RNA through the microRNA pathway. Future studies may include the possibility of integrating the intron-containing multiple shRNA constructs into the chicken PGCs with techniques such as direct injection or PGC culture to produce a transgenic bird [38]. Taken together, the expression of shRNAs without the need for exogenous promoter elements, would substantially benefit a broad range of applications of RNAi technology.

## Data Availability

Data is contained within the article or supplementary material.

## Supplementary Materials

The following supporting information can be downloaded at: https://www.mdpi.com/article/10.3390/mps5010018/s1, Table S1. Synthesised oligonucleotides used in this study, Table S2. siRNA sequences used in this study. Figure S1. Mfold schematics of shRNAs with traditional Brummelkamp (BK) loop and miR30 adapted structures. The PB1 siRNA sequence was embedded in the miR30 structure (miR30-PB1) with both the sense and antisense sequence connected by a loop. The natural miRNA sequence is shown alongside of miR30-PB1. The incorporated PB1 sequence within the miR30-PB1 shRNA is highlighted with a red line. Figure S2. The predicted RNA secondary structures of a section of the natural intron 5 of PANK1 without (left) and with shRNA (right). The miR107 sequence is highlighted in red colour and the green arrow indicates the inserted miR30-PB1 shRNA within the intron. Figure S3. The selected loop sequences downstream of miR107 to be replaced with the miR30-PB1, miR30-NP and miR30-NS are highlighted in red. The comparison of the predicted secondary structure of the natural intron (top) and intron with three shRNAs (bottom) is also shown. Figure S4. The selected intron 5 region of the PANK1 gene to insert three shRNAs. The three selected sites downstream to miR-107 are highlighted in red (top) and the intron sequence with PB1, NP and NS shRNA was provided (bottom). The intron containing three shRNAs (466 bp) was oligo-synthesised and used to clone into pGI vector.
