# Supplementary material for: Harnessing Intronic microRNA Structures to Improve Tolerance and Expression of shRNAs in Animal Cells
Source: Methods Protoc. 2022 Feb 10;5(1):18. doi: 10.3390/mps5010018 (PMC8879667; doi:10.3390/mps5010018)
Supplement: Supplementary file 1 [file mps-05-00018-s001.zip › mps-1532894-supplementary.pdf]

## Supplementary figure legends

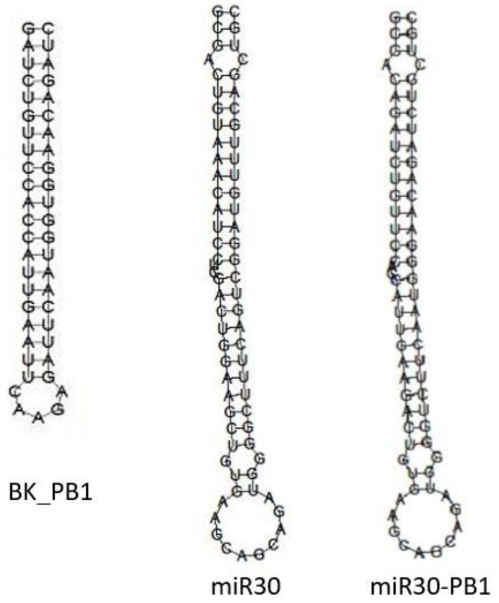


**Figure S1.** Mfold schematics of shRNAs with traditional Brummelkamp (BK) loop and miR30 adapted structures. The PB1 siRNA sequence was embedded in the miR30 structure (miR30-PB1) with both the sense and antisense sequence connected by a loop. The natural miRNA sequence is shown alongside of miR30-PB1.

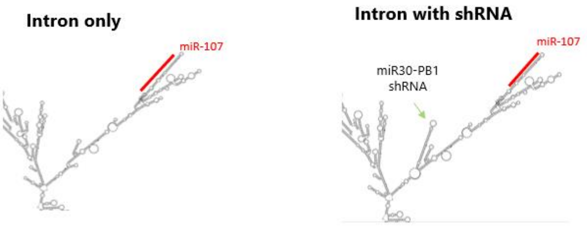


**Figure S2.** The predicted RNA secondary structures of a section of the natural intron 5 of PANK1 without (left) and with shRNA (right). The miR107 sequence is highlighted in red colour and the green arrow indicates the inserted miR30-PB1 shRNA within the intron. .

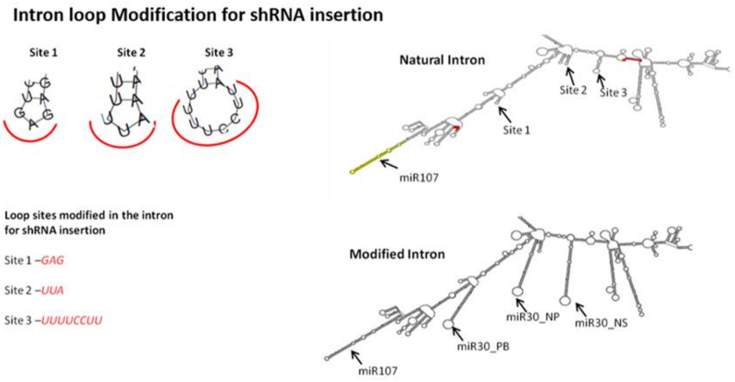


**Figure S3.** The selected loop sequences downstream of miR107 to be replaced with the miR30-PB1, miR30-NP and miR30-NS are highlighted in red colour. The comparison of the predicted secondary structure of the natural intron (top) and intron with three shRNAs (bottom) is also shown.

**Selection of three sites for shRNA insertion within intron 5:**

Site 1

GGATCCATAGCATTGTATGGAGGACGGTAGTTTGATGTATTAAGTACGCTTCACATCCTAGACAACATCTGAGAGATG
AATTTATTTTGTGGCTTTAACAAAAGAGAACTTAATTCAGTGGCTTTACATGTTTTTTTTAAAAAGGCTTCAAATGTAG
CATATTGTTGCAAGGCAGCTACTTTCTAGATAGTGTTATTTTTTTCCTTAAATAAAGCTATGTTCTGATAAATACTCCAG
ACACCTACATTGTTTTTGTGAGGTACC

Site 3 Site 2

**Modified intron 5 with three shRNAs:**

Site 1 with PB1 shRNA

GGATCCATAGCATTGTATGGAGGACGGTAGTTTGATGTATTAAGTACGCTTCACATCCTAGACAACATCTGCGCGATC
TGTTCCACTGCATTGAAGACTGTGAAGCAGCAGATGGGGTCTTCAATGGTGGAACAGATCTTGCAGATGAATTTATT
TTGTGGCTTTAACAAAAGAGAACTTAATTCAGTGGCTTTACATGTTTTTTGCGAATCTTATTTCTTGCCGGAGACAAC
TGTGAAGCAGCAGATGGGGTTGTCTCCGAAGAAATAAGATCTGCAAAAGGCTTCAAATGTAGCATATTGTTGCAAG
GCAGCTACTTTCTAGATAGTGTTATTTGCGAAATGGAATGATATCACACAGTTCCTGTGAAGCAGCAGATGGGGGAA
CTGTGTTATCATTCCATTCTGCAAATAAAGCTATGTTCTGATAAATACTCCAGACACCTACATTGTTTTTGTGAGGTACC

Site 3 with NS shRNA Site 2 with NP shRNA

**Figure S4.** The selected intron 5 region of the PANK1 gene to insert three shRNAs. The three selected sites downstream to miR-107 are highlighted in red (top) and the intron sequence with PB1, NP and NS shRNA was provided (bottom). The intron containing three shRNAs (466 bp) was oligo-synthesised and used to clone into pGI vector.

**Table S1.** Synthesised oligonucleotides used in this study.

| Name | sequence |
|---|---|
| GFP_ fwd | ACTGTCGACATGGTGAGCAAGGGCGAGG |
| GFP_ rwd | ACTGCGGCCGCTTACTTGTACAGCTCGTCC |
| SmaI_fwd | GACCCGGGCCGAGGTGAAG |
| SmaI_rev | CTTCACCTCGGCCCGGGTC |
| Intron_107_fwd | GTAAGATGGTGTATTTACTATGAGC |
| Intron_107_rev | CTGTAAAATTGAAAAGCAG |
| BamHI_int_fwd | ACTAGATGGGATCCATAGC |
| BamHI_int_rev | GCTATGGATCCCATCTAGT |
| KpnI_int_fwd | TGTGAGGTACCGCTCTAAC |
| KpnI_int_rev | GTTAGAGCGGTACCTCACA |

| | |
|---|---|
| BK_ PB1_fwd | GATCGATCTGTTCCACCATTGAATTCAAGAGATTC AATGGTGGAACAGATCGTAC |
| BK_ PB1 _rev | GATCTGTTCCACCATTGAATCTCTTGAATTCAATGG TGGAACAGATC |
| miR30_ PB1_fwd | GATCGCGACAGATCTGTTCCACCATTGAAGACTGT GAAGCAGCAGATGGGGTCTTCAATGGGAACAGAT CTGCTGCGTAC |
| miR30_ PB1_rev | GCAGCAGATCTGTTCCCATTGAAGACCCCATCTGC TGCTTCACAGTCTTCAATGGTGGAACAGATCTGTCG C |
| PB1_luc_fwd | CTCGAGGGAGATCATTCGAGCTGAAG |
| PB1_luc_rev | GCGGCCGCCCGTCTGAGTTCTTCAATGG |
| NP_luc_fwd | GCGCTCGAGACCAAATGAGAATCCAGCAC |
| NP_luc_rev | AGCGCGGCCGCAATTGTCGTACTCCTCTGCA |
| NS_luc_fwd | ATGCTCGAGGTACTCTCGGTCTGGACATC |
| NS_luc_rev | ATCGCGGCCGCTTCCCGCCATTTCTCGTTTC |
| miR-PTA | GAGGCGAGCACAGAATTAATACGACTCCATATAG GTTTTTTTTTTTTVN |
| PAM-URP | GAGGCGAGCACAGAATTAATACGAC |
| miR107 | GCAGCAGCATTGTACAGGGCT |
| miR26a | GCCTTCAAGTAATCCAGGATAGG |
| PB1 | CGCTTCAATGGTGGAACAGATC |
| 5S | TGGGAATACCGGGTGCTGT |
| Poly-A_fwd | ACTGCGGCCGCGACTCTAGATCA |
| Poly-A_rev | ACTGAATTCACGCTTACAATTTACGC |

**Table S2.** siRNA sequences used in this study.

| Name | Sequence (5′ – 3′) |
|---|---|
| PB1-2257 | GATCTGTTCCACCATTGAA |
| NP | GATCTGTTCCACCATTGAA |
| NS1/NEP | AATGGAATGATAACACAGT |
